# Genotyping of ancient *Mycobacterium tuberculosis* strains reveals historic genetic diversity

**DOI:** 10.1098/rspb.2013.3236

**Published:** 2014-04-22

**Authors:** Romy Müller, Charlotte A. Roberts, Terence A. Brown

**Affiliations:** 1 Manchester Institute of Biotechnology, Faculty of Life Sciences, University of Manchester, 131 Princess Street, Manchester M1 7DN, UK; 2 Department of Archaeology, Durham University, South Road, Durham DH1 3LE, UK

**Keywords:** tuberculosis, ancient DNA, strain typing, single nucleotide polymorphisms, mixed infection

## Abstract

The evolutionary history of the *Mycobacterium tuberculosis* complex (MTBC) has previously been studied by analysis of sequence diversity in extant strains, but not addressed by direct examination of strain genotypes in archaeological remains. Here, we use ancient DNA sequencing to type 11 single nucleotide polymorphisms and two large sequence polymorphisms in the MTBC strains present in 10 archaeological samples from skeletons from Britain and Europe dating to the second–nineteenth centuries AD. The results enable us to assign the strains to groupings and lineages recognized in the extant MTBC. We show that at least during the eighteenth–nineteenth centuries AD, strains of *M. tuberculosis* belonging to different genetic groups were present in Britain at the same time, possibly even at a single location, and we present evidence for a mixed infection in at least one individual. Our study shows that ancient DNA typing applied to multiple samples can provide sufficiently detailed information to contribute to both archaeological and evolutionary knowledge of the history of tuberculosis.

## 1. Introduction

Tuberculosis (TB) has caused millions of deaths throughout history and is still a major burden in many parts of the world. Especially in the seventeenth–nineteenth centuries AD, TB was highly prevalent throughout Europe, urbanization facilitating spread of the disease in overcrowded environments [1]. The improved living standards in the late nineteenth century AD led to a decline in incidence rates and a further drop was achieved by the use of antibiotics in the mid-twentieth century [1,2]. Since its re-emergence in the 1980s, however, TB has once again become one of the leading infectious diseases, causing morbidity and mortality in all parts of the world, with an estimated 1.4 million deaths in 2011 [3].

TB is caused by the members of the *Mycobacterium tuberculosis* complex (MTBC), with *M. tuberculosis* being the most common infecting species in humans. The MTBC also comprises the human pathogens *M. africanum* and *M. canettii* as well as the primarily animal infecting species *M. bovis*, *M. microti*, *M. pinnipedii* and *M. caprae*, all of which have been identified as causative agents of TB in humans [4–8]. The continuing appearance of antibiotic-resistant MTBC strains has stimulated interest in the evolutionary history of TB, in particular the possible coevolution between MTBC lineages and human populations [9]. Modern genetic data indicate that the MTBC may have coexisted with humans for at least 15 000 years [10–14], and archaeological evidence suggests that it has afflicted humankind since the Neolithic [15–18]. Throughout the past two decades, studies of ancient DNA (aDNA) in archaeological remains have begun to contribute to our understanding of the evolutionary history of the MTBC, several publications reporting the presence of MTBC aDNA in human remains, and some attempting to classify the infecting MTBC strains based on the identities of various genetic markers [17–28].

Several methods are used in clinical research to classify MTBC isolates into groups of related strains, including IS6110 restriction fragment length polymorphism (IS6110 RFLP) [29], mycobacterial interspersed repetitive unit-variable number tandem repeat (MIRU-VNTR) typing [30,31], spacer oligotyping (spoligotyping) [32], targeting of large sequence polymorphisms (LSPs) [33–35] and typing of single nucleotide polymorphisms (SNPs) [13,36–39]. RFLP analysis of IS6110, MIRU-VNTR typing and spoligotyping are less suitable for phylogenetic analyses as convergent evolution may result in homoplasy of the targeted loci [40,41]. Spoligotyping has been applied in studies of ancient TB [17–24] but its use has been questioned [42], not just because of its limited phylogenetic value but also because of its methodological problems when highly degraded aDNA is being analysed. LSPs and SNPs are considered to be the most suitable markers for strain identification and phylogenetic analysis, although they are not exempt from convergent evolution [43,44]. Early sets of SNPs were discovered by analysing selected genes or comparing genome sequences from only few strains, thereby not accessing a large part of the global variation [45]. More recently, de novo sequencing of 89 genes in 108 strains from all parts of the world identified 488 SNPs and resulted in a detailed phylogeny of the MTBC [46]. Whole genome comparison of 22 globally representative, mainly newly sequenced strains subsequently revealed 9037 unique SNPs and further resolved the phylogeny of the MTBC [47], and 34 167 SNPs identified from 259 strains have been used to study the coevolutionary history of *M. tuberculosis* and prehistoric human populations [14]. Targeting such large sets of loci is not feasible in aDNA studies if conventional PCR procedures are applied because the amount of extract available for analysis is usually very limited. These methodological constraints can be overcome by next generation sequencing (NGS), which is now being adopted in human aDNA studies and has recently been applied to historic strains of plague [48], TB [49] and leprosy [50]. However, NGS methods have their own limitations, requiring relatively large amounts of aDNA, being computationally intensive, and suffering from missing data owing to the absence of sequence reads covering particular SNPs, even ones that can be typed in the same sample by conventional PCR [49].

In this study, we show that an informative comparison of MTBC strains in archaeological human bone and dental samples is possible by conventional PCR of eleven SNPs and two LSPs (table 1). These markers enable MTBC strains to be classified into principal genetic groups (PGGs) 1–3 [36], lineages I–IV and *M. bovis* [38], SNP cluster groups (SCGs) 1–7 [39], ‘modern’ *M. tuberculosis* [33] and the Euro-American lineage of modern *M. tuberculosis* [34,35]. Five additional SNPs [51] allow a more precise classification of strains of the Euro-American lineage. We typed these markers in British and other European archaeological bone and dental samples from skeletons dated to the second–nineteenth centuries AD, revealing historic variations in genotype identities.

**Table 1.** Loci targeted in this study in order to classify ancient MTBC strains.

locus type	locus^a^	classification into	references
SNP	*gyrA*^284^	PGGs 1–3	[36]
	*katG*^1388^		
	*oxyR*^37^	lineages I–IV and *M. bovis*	[38]
	*oxyR*^285^		
	*rpoB*^2646^		
	*rpoB*^3243^		
	*leuB* (3352929)	SNP cluster groups 1–7	[39]
	*qcrB* (2460626)		
	*recN* (1920118)		
	Rv0083 (92197)		
	Rv2802c (3111473)		
LSP	TbD1	ancient/modern *M. tuberculosis*	[33]
	*pks15/1*	Euro-American lineage	[34,35]

^a^Numbers in parentheses denote the nucleotide position in H37Rv, as given in Filliol *et al*. [39].

## 2. Material and methods

Thirty-four bone and dental samples were selected based on the positive outcomes of PCRs directed at the IS6110 and IS1081 insertion sequences [52], which are looked on as specific for the MTBC group of bacteria. The samples come from skeletons dated to the second–nineteenth centuries AD and most but not all displayed lesions specific or non-specific for TB (electronic supplementary material, table S1). We have previously reported NGS and conventional SNP typing results for one of these samples, St George's Crypt 4006 [49].

Samples were taken under clean conditions by personnel wearing forensic suits, hair nets, face masks and sterile gloves, and stored in sterile plastic bags under dry conditions. Work was performed at the University of Manchester and the Complutense University of Madrid. The aDNA facility at the University of Manchester comprises independent, physically isolated laboratories for extraction and PCR set-up, each with an ultrafiltered air supply maintaining positive displacement pressure. DNA extractions were prepared in a Class II biological safety cabinet, and PCRs were set up in a laminar flow hood. Surfaces were sterilized by UV irradiation and regularly cleaned with 5% bleach and 70% ethanol. All equipment was treated with DNA-Away (Molecular Bioproducts) and tubes, pipettes and aqueous solutions were UV irradiated (254 nm, 120 000 µJ cm^−2^) for at least 10 min before use. Personnel wore protective clothing at all times, including forensic suits, face masks, hair nets, goggles and two pairs of sterile gloves. Work in Madrid was also carried out in physically separated laboratories for DNA extraction and PCR set-up, UV irradiated both before and after use. Surfaces and laboratory equipment were regularly cleaned with bleach. Personnel wore disposable forensic suits, face masks, caps, glasses, shoe covers and gloves. All reagents and consumables were DNase and RNase free. All procedures were carried out in a laminar flow cabinet, UV irradiated and cleaned with bleach before use. DNA extractions were accompanied by two blanks (extraction without skeletal material) per five samples (Manchester) or one blank per seven samples (Madrid). A set of 5–7 PCRs was always accompanied by at least two PCR blanks (set up with water instead of DNA extract).

Samples were prepared by removing approximately 1–2 mm of the outer surface of the bone mechanically, followed by UV irradiation (254 nm, 120 000 µJ cm^–2^) for 2 × 5 min, with 180° rotation between the two exposures, and subsequent crushing of the bone into powder. Each tooth was cleaned externally by placing it for 5 min in a small beaker containing 5% bleach, without allowing bleach to enter the root canal. The tooth was then dried with a paper towel, placed in a second beaker and rinsed in Millipore water, again avoiding entry of water into the root canal. After drying, a 37% phosphoric acid etching solution was applied to the tooth surface, left for 1 min and wiped off. The tooth was rinsed in Millipore water, dried for 10 min, and dentine powder collected using a dental pick.

At least two extractions were prepared for each sample, using 250 mg of bone or 50–100 mg of dentine powder. Extractions at Manchester used the method described by Bouwman & Brown [53] and/or a protocol based on Rohland & Hofreiter [54] and Rohland *et al*. [55], previously described by Bouwman *et al*. [49]. A subset of samples was re-extracted at Madrid using the latter method only. PCRs were directed at up to 16 SNPs and two LSPs (table 1; electronic supplementary material)*.* PCRs were set up in a final volume of 30 µl, comprising 3–5 µl of DNA extract, 1× AmpliTaq Gold PCR Master Mix (Applied Biosystems), 400 nM each primer and 1% BSA. Cycling conditions were: 7 min at 95°C; followed by 45 cycles of 1 min at *x*°C and 1 min at 94°C; followed by 10 min at 72°C, where *x°*C is the primer-specific annealing temperature (electronic supplementary material, table S2). For primers with an annealing temperature less than or equal to 60°C, a three-temperature PCR was set up, with each annealing step at *x*°C followed by a 1 min extension at 72°C. All PCR products were examined by electrophoresis in 2% agarose gels, purified either from the gel or directly using Qiaquick columns (Qiagen) and subsequently cloned into *Escherichia coli* XL1-Blue competent cells (Agilent) using the CloneJet PCR cloning kit (Fermentas). Colony PCR was performed in 20 μl comprising 1× *Taq* buffer (New England Biolabs), 200 nM each primer, 200 μM dNTPs and 0.625 units *Taq* DNA polymerase (New England Biolabs), with cycling at: 95°C for 3 min; 30 cycles of 30 s at 94°C, 30 s at 60°C, 1 min at 72°C; 10 min at 72°C. PCR products were then sequenced (GATC Biotech, Cologne) and sequences aligned with the *M. tuberculosis* H37Rv reference sequence using Geneious v. 6.0.3 (http://www.geneious.com/).

To compare our results with 21 extant MTBC strains, targeted regions available from the six samples that provided unambiguous typing results were concatenated and aligned with the equivalent concatenated regions of the extant MTBC strains. For Auldhame 43, this procedure was repeated for all available sequences, including the four additional SNPs taken from Abadia *et al*. [51]. Neighbour-joining trees were created using MEGA v. 5.2.1 [56] and visualized with Dendroscope v. 3.2.4 [57].

## 3. Results

We analysed 34 samples from 31 individuals from 22 archaeological sites and one pathological reference collection (electronic supplementary material, table S1) for 11 SNPs and two LSPs (table 1). We obtained data for ten samples from seven archaeological sites (electronic supplementary material, table S3), enabling us to classify the infecting MTBC strains according to their SNP identities, the presence/absence of the TbD1 locus, and/or the presence of deletions within *pks15/1* (table 2). Six of the samples (Auldhame 43, Saint Amé 20, St George's Crypt 4006, St Peter's Church 1390, St Peter's Collegiate Church 62, Whitefriars 10466) provided sufficient information, reproducible with two independent extracts, to assign the infecting strains to both their PGGs [36] and SCGs [39]. Two other samples, St Peter's Collegiate Church 28 and St George's Crypt 5003, gave incomplete results that enabled a PGG and/or SCG to be assigned from one extract but not confirmed with the second extract. Ambiguous results were obtained with two additional samples. Whilst Whitefriars 657 was identified as containing a SCG 6 strain, the *gyrA*^284^ SNP was typed as a C in one extract and G in the other, suggesting either PGG 2 or 3. With Ashchurch 705, clone sequences from the first extract gave a mixture of C and G at this position, and the *qcrB* SNP, which distinguishes SCG 3 from SCG 4, could be typed with only one of the two extracts (electronic supplementary material, table S3).

**Table 2.** Summary of SNP and LSP data.^a^ —, no result obtained; n.d., not done; TbD1^−^, deletion of TbD1 has occurred; Δ7 bp, 7 bp deletion in *pks15/1* has occurred.

sample ID	sample date	TbD1	*pks15/1*	PGG	lineage	SCG
Ashchurch 705^b^	129–317 calAD	TbD1^−^/TbD1^−^	Δ7 bp/Δ7 bp	^2^/_3_/2	I or II/–	3/3 or 4
Auldhame 43^b^	1280–1394 calAD	TbD1^−^/n.d.	Δ7 bp/Δ7 bp	2/2	II/–	5/5
Saint Amé 20	16th–18th centuries AD	–/n.d.	–/Δ7 bp	3/3	not IV/not III	6/6
St George's Crypt 4006^b^	mid-19th century AD	TbD1^−^/TbD1^−^	Δ7 bp/Δ7 bp	3/3	II/II	6/6
St George's Crypt 5003	mid-19th century AD	–/TbD1^−^	Δ7 bp/–	–/–	not IV/–	6/–
St Peter's Church 1390^b^	1016–1155 calAD	TbD1^−^/n.d.	Δ7 bp/Δ7bp	2/2	I or II/–	3/3
St Peter's Collegiate Church 28	19th century AD	TbD1^−^/n.d.	Δ7 bp/–	3/–	I or II/–	6/–
St Peter's Collegiate Church 62^b^	19th century AD	TbD1^−^/TbD1^−^	Δ7 bp/Δ7 bp	2/2	I or II/II	4/4
Whitefriars 657	18th–19th centuries AD	–/n.d.	Δ7 bp/–	2/3	I or II/–	6/6
Whitefriars 10466^b^	18th–19th centuries AD	TbD1^−^/n.d.	–/Δ7 bp	2/2	I or II/–	4/4

^a^Results listed as first/second extraction.

^b^Second extraction performed in Madrid, except for TbD1.

The *oxyR* and *rpoB* PCRs used to assign strains to lineages I–IV [38] were less successful owing to lack of reproducibility and amplification of non-specific *rpoB*^3243^ targets. Nine of the 10 samples could be assigned to lineages I or II, but distinction between these lineages, which requires *rpoB*^3243^, was only possible for Auldhame 43, St Peter's Collegiate Church 62 and St George's Crypt 4006, each of which was identified as lineage II based on results with one or both extracts. St George's Crypt 5003 could only be identified as not lineage IV. The *pks15/1* PCR revealed a 7 bp deletion for each of the 10 samples, suggesting that the strains belong to the Euro-American lineage, and amplification of the region flanking TbD1 was achieved for eight samples, again indicating that these are modern *M. tuberculosis* strains.

**Figure 1.**
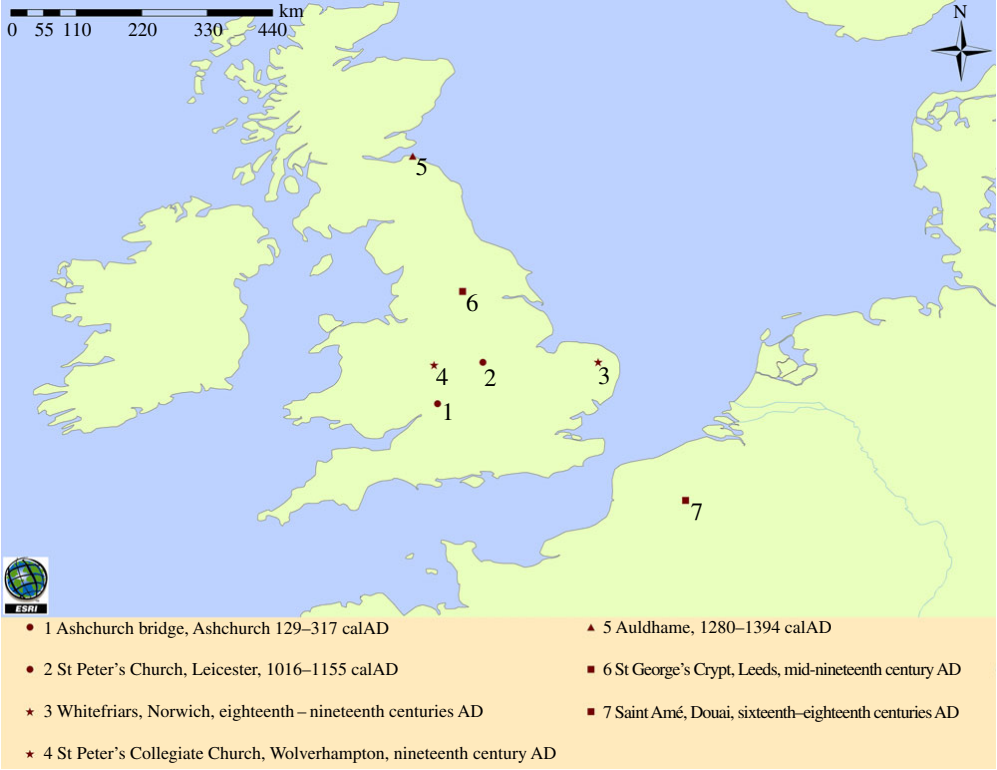
Location of the sites for the 10 samples for which we report strain data. Circles, location of a strain belonging to PGG 2 and SCG 3; triangle, PGG 2 and SCG 5 strains; squares, PGG 3 and SCG 6 strains; stars, sites for which both PGG 2 and PGG 3 as well as SCG 4 and SCG 6 strains were identified. Note that evidence for an additional second strain belonging to PGG 3 was obtained for the sample from the individual from Ashchurch. (Online version in colour.)

**Figure 2.**
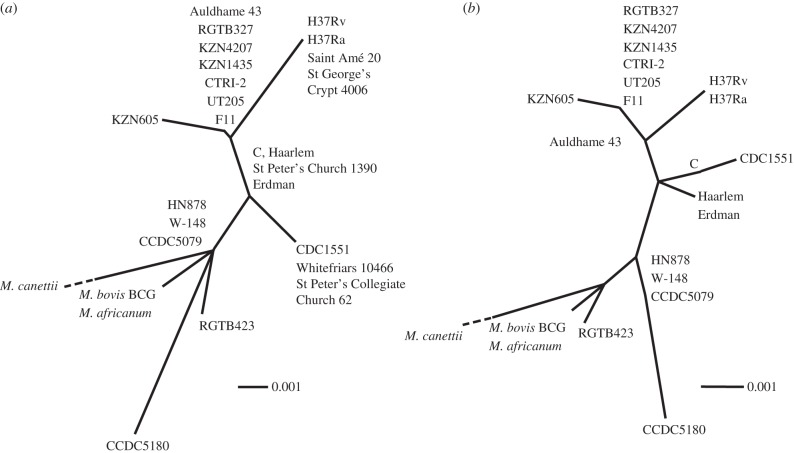
(*a*) Neighbour-joining tree comparing the concatenated sequences of eight regions (478 bp—*gyrA*, *katG*, *leuB*, *oxyR*^37^, *pks15/1*, *qcrB*, *rpoB*^2646^ and Rv0083) obtained from a total of six samples (Auldhame 43, Saint Amé 20, St Peter's Church 1390, St Peter's Collegiate Church 62, St George's Crypt 4006 and Whitefriars 10466) with the equivalent regions of 21 extant MTBC strains. These extant MTBC strains are *M. tuberculosis* strains H37Rv (National Center for Biotechnology Information reference sequence NC_000962.2), H37Ra (NC_009525.1), ATCC35801 str. Erdman (AP012340.1), CCDC5079 (NC_017523.1), CCDC5180 (NC_017522.1), CDC1551 (NC_002755.2), CTRI2 (NC_017524.1), F11 (NC_009565.1), KZN605 (NC_018078.1), KZN1435 (NC_012943.1), KZN4207 (NC_016768.1), RGTB327 (NC_017026.1), RGTB423 (NC_017528.1), UT205 (NC_016934.1) and HN878 (CM001043.1) as well as *M. bovis* bacillus Calmette–Guérin str. Mexico, (NC_016804.1), *M. africanum* GM041182 (NC_015758.1) and *M. canettii* CIPT140010059 (NC_015848.1). Further strain data from whole genome shotgun sequencing projects was available from the Broad Institute (*M. tuberculosis* comparative sequencing project, Broad Institute of Harvard and MIT (http://www.broadinstitute.org/)) for: *M. tuberculosis* C (GenBank accession number AAKR00000000), *M. tuberculosis* Haarlem (AASN00000000) and *M. tuberculosis* W-148 (ACSX00000000.1). (*b*) Neighbour-joining tree comparing the concatenated sequences of 16 regions (1054 bp) obtained from Auldhame 43 with the equivalent regions of the 21 extant MTBC strains listed in (*a*). Bootstrap values were weak for both trees, as expected due to the small character set.

All but one of the 10 samples for which we obtained strain data came from British excavation sites spanning the second–nineteenth centuries AD, the one exception coming from sixteenth–eighteenth centuries AD Douai in northern France (figure 1). The relationships between these strains and modern ones are depicted as a neighbour-joining tree in figure 2*a*. Additional targeting of five of the SNPs reported by Abadia *et al*. [51] was attempted with one extract of Auldhame 43, a member of SCG 5. Four SNPs were typed, enabling more accurate resolution of the position of Auldhame 43 compared to the extant MTBC strains (figure 2*b*).

## 4. Discussion

We obtained sufficient SNP and/or LSP data to classify the *M. tuberculosis* strains present in 10 of the 34 bone and dental samples that we studied. Our results clearly show that, at least during the eighteenth–nineteenth centuries AD, strains of *M. tuberculosis* belonging to different genetic groups were present in Britain at the same time, possibly even at a single location. Within this time period, we discovered PGG 2/SCG 4 strains in individuals at sites in Norwich, eastern England (Whitefriars 10466), and Wolverhampton, central England (St Peter's Collegiate Church 62), and a PGG 3/SCG 6 strain at Leeds, northern England (St George's Crypt 4006). Strains of both types might even have coexisted in the same local areas, as a second Wolverhampton individual (St Peter's Collegiate Church 28) had a PGG 3/SCG 6 strain, and a second individual from Norwich (Whitefriars 657) had a PGG ^2^/_3_/SCG 6 strain. Both of these identifications are, however, tentative (at least according to the strict technical regime that we adopted) because they were not fully reproducible. Only one extract from St Peter's Collegiate Church 28 gave results, and the PGG classification for Whitefriars 657 was ambiguous, PGG 2 being identified with the first extraction and PGG 3 with the second.

One explanation of the ambiguous Whitefriars 657 result is that this individual was co-infected with two strains of *M. tuberculosis*, one strain belonging to PGG 2 and the other to PGG 3. Based on the presence of a T at Rv0083, in both extracts, the infecting strains could further be assigned to SCG 6. However, SNP typing success with the second extract was limited (table 2; electronic supplementary material, table S3), and for this reason we look on the individual from Whitefriars (657) as having possible but not definite mixed infection, i.e. TB caused by two different strains. A second, more convincing example of mixed infection was presented by the Roman sample Ashchurch 705. One of the extracts of this sample gave both possible nucleotides for *gyrA*^284^ (C and G), indicating an infection with both PGG 2 and PGG 3 strains. Owing to the type of polymorphism, we exclude the disparity as resulting from a miscoding lesion [58–60], and there was no evidence of contamination (electronic supplementary material). Mixed infection has been reported with patients today, either as concurrent infections with multiple MTBC strains or as exogenous re-infections [61]. The presence of a mixed infection in Ashchurch 705 also indicates that both PGG 2 and PGG 3 strains coexisted in southwest Britain during the second–fourth centuries AD. Since the second extract for Ashchurch 705 revealed a PGG 2 strain only, we assume that the SCG genotype obtained from both extracts derived from this PGG 2 strain. However, we cannot establish if the PGG 3 strain displayed the same SCG genotype as the PGG 2 strain.

Filliol *et al*. [39] have suggested that, of the four SCGs 3–6, SCG 3 preceded SCG 5 with the latter followed by SCG 4 and SCG 6 appearing lastly. Although our sample size is small, the results are consistent with this succession of SCG types. The three oldest samples that yielded genotype data were from skeleton 705 from Ashchurch (129–317 calAD) and St Peter's Church 1390 (1016–1155 calAD), both of whom had strains belonging to PGG 2/SCG 3, and skeleton 43 from Auldhame (1280–1394 calAD), which was identified as PGG 2/SCG 5. The remaining six British samples, dated to the eighteenth–nineteenth centuries AD, were from individuals with either PGG 2/SCG 4 or PGG 3/SCG 6, with skeleton 657 from Whitefriars possibly also harbouring a PGG 2 strain. Additionally, a sixteenth–eighteenth century AD individual from Douai, northern France (Saint Amé 20), was shown to contain a PGG 3/SCG 6 strain.

The markers we typed do not distinguish the a and b subgroups of SCG 6. However, we have previously shown by NGS genotyping that skeleton 4006 from St George's Crypt had a SCG 6b strain [49]. This is the same SCG as the MTBC reference strain H37Rv [39], which was first isolated at the beginning of the twentieth century from a North American patient [62]. Our results therefore indicate that strains similar to H37Rv might have been present in continental Europe in the sixteenth–eighteenth centuries AD and geographically dispersed in eighteenth–nineteenth centuries AD Britain.

Strains belonging to SCG 3b, 3c, 4 and 5 fall into PGG 2 as they harbour a polymorphism at katG^1388^ but not *gyrA*^284^ [39]. By contrast, SCG 6 strains display polymorphisms at both *katG*^1388^ and *gyrA*^284^ and are therefore classified as PGG 3 [39]. Further analyses will be necessary in order to identify whether skeletons from Ashchurch (705) and St Peter's Church (1390) indeed had strains belonging to SCG 3b or 3c as suggested by the PGG 2 determination or if they had strains belonging to subgroup SCG 3a which is primarily found within PGG 1 [39], with possible exceptions [63]. PGGs 2 and 3 (and likewise their associated SCGs) are also known to comprise lineage II strains, as identified by a polymorphism at *rpoB*^3243^ [38], and to lack the *M. tuberculosis*-specific deletion TbD1 [33] as well as a 7 bp region within *pks15/1* [34]. Deletion of the 7 bp region was revealed for all 10 of our samples, with eight of them also shown to lack TbD1, classifying them as strains belonging to the Euro-American clade of modern *M. tuberculosis*. While deletion of TbD1 has previously been reported in an individual from Britain dating to approximately 2200 years BP [25], our results for Ashchurch 705 now additionally disclose that the deletion of the 7 bp region within *pks15/1* had already taken place by the second–fourth centuries AD in Britain.

By typing four of the SNPs reported by Abadia *et al*. [51], we showed that the infecting SCG 5 strain found in the skeleton of Auldhame 43 is a member of the group that is ancestral to extant strains of the Latin-American Mediterranean clade within the Euro-American lineage. The detection of a SCG 5 strain at Auldhame (a coastal settlement east of Edinburgh, Scotland) but a SCG 3 strain at Leicester only about 100–200 years earlier raises the possibility that the Auldhame strain was not introduced into the human population in Scotland from southern parts of Britain but from Scandinavia instead. The first archaeological evidence of TB in Scotland [64] pre-dates Viking invasions in the late eighth century AD and Scandinavian contact in subsequent centuries [65], and stable isotope analysis of samples from individuals from the Auldhame site has indicated that the skeletal population itself is most likely composed of local individuals [66]. Nevertheless, introduction of (new) TB strains via this route is a possible scenario as the osteological evidence suggests the presence of TB in Scandinavia during the Iron Age (fifth–first centuries BC) as well as the medieval period (1050–1536 AD) [1,67–69].
